# A Review of fMRI Affective Processing Paradigms Used in the Neurobiological Study of Posttraumatic Stress Disorder

**DOI:** 10.1177/2470547019829035

**Published:** 2019-02-25

**Authors:** Alyson M. Negreira, Chadi G. Abdallah

**Affiliations:** 1 Clinical Neurosciences Division, United States Department of Veterans Affairs, National Center for Posttraumatic Stress Disorder, VA Connecticut Healthcare System, West Haven, CT, USA; 2 Department of Psychiatry, Yale University School of Medicine, New Haven, CT, USA

**Keywords:** Posttraumatic stress disorder, affective processing, functional magnetic resonance imaging, neurobiology, review

## Abstract

Posttraumatic stress disorder is a chronic and debilitating psychiatric disorder with a complex clinical presentation. The last two decades have seen a proliferation of literature on the neurobiological mechanisms subserving affective processing in posttraumatic stress disorder. The current review will summarize the neuroimaging results of the most common experimental designs used to elucidate the affective signature of posttraumatic stress disorder. From this summary, we will provide a heuristic to organize the various paradigms discussed and report neural patterns of activations using this heuristic as a framework. Next, we will compare these results to the traditional functional neurocircuitry model of posttraumatic stress disorder and discuss biological and analytic variables which may account for the heterogeneity within this literature. We hope that this approach may elucidate the role of experimental parameters in influencing neuroimaging findings.

Posttraumatic stress disorder (PTSD) is a debilitating psychiatric condition which develops due to the impediment of recovery after the experience of a traumatic event. Currently, PTSD is viewed as having a complex and heterogeneous clinical presentation, spanning four symptom clusters: (1) intrusive memories, distressing dreams, or flashbacks; (2) persistent avoidance of trauma reminders; (3) negative changes in thoughts and mood; and (4) heightened arousal reactivity.^1^ Although the experience of a traumatic event throughout the lifetime is unfortunately common,^2,3^ only a minority of individuals will go on to develop PTSD. It is estimated that 6.8% of individuals in the general population will meet criteria for PTSD;^4^ however, this rate substantially increases in populations with greater trauma exposure.^5^ For example, a study conducted by the Congressional Budget Office^6^ found that 21% of individuals from overseas contingencies operations (OCO) in Iraq and Afghanistan met criteria for PTSD. Furthermore, the Veterans Health Administration’s average cost of treating OCO veterans with a PTSD diagnosis was approximately four to six times greater than those not carrying the same diagnosis.^6^ Despite the prevalence and substantial burden, the neurobiology and concomitant neuropharmacological treatments for PTSD are not well understood. As such, there is an increasing need to identify the neural pathogenesis of this disorder.

One approach in understanding the neurobiology of PTSD has been through functional neuroimaging techniques. Indeed, the last two decades have seen a rapid growth in the utilization of functional magnetic resonance imaging (fMRI), positron emission tomography (PET), and single-photon emission computed tomography (SPECT) in the neurobiological inquiry of PTSD. Commonly, these functional techniques employ paradigms designed to elicit re-experiencing symptoms,^7^ which were historically viewed as the defining feature of PTSD.^8,9^ Also known as “symptom provocation” paradigms, these studies focused on affective processes and aimed to elicit neural activity in response to trauma-related reminders, such as idiographic traumatic scripts, images, and sounds. In addition to findings from non-human animal studies, the results of symptom provocation paradigms put forth a prevailing and elegant functional neurocircuitry model (FNM) of PTSD first proposed by Rauch et al.^7^ The FNM views PTSD symptomology through the lens of a fear-conditioning framework. Specifically, it was contended that the amygdala is hyperactivated in individuals with PTSD, contributing to heightened processing of fearful and threatening stimuli. Next, the medial prefrontal cortex (mPFC), including the anterior cingulate cortex (ACC), subcallosal cortex, and medial frontal gyrus, are found to be hypoactive in individuals with PTSD, resulting in inappropriate persistent fear of trauma and non-trauma-related stimuli. Finally, the hippocampus is proposed to functional abnormally in individuals with PTSD, leading to difficulty with adaptive fear learning and extinction.^10^

Although the FNM has reigned predominate as the most referenced neurobiological model of PTSD, several other salient models should be noted. The triple network model proposes aberrant neuronal functioning of the central executive, salience, and default mode networks, which may lead to symptoms of multiple psychopathologies.^11^ When applied to the neurobiology underscoring the clinical manifestation of PTSD, Patel et al.^12^ contend that deficits in the central executive network, specifically that of the dorsolateral PFC (dlPFC) and lateral areas of the parietal lobe, are underutilized in individuals with PTSD, whereas the precuneus is reliably over recruited. In contrast to these findings, Patel et al.’s assert that the salience network exhibits greater activation in individuals with PTSD, specifically in the anterior insula and dorsal ACC. Finally, the default mode network, specifically the mPFC, posterior cingulate cortex, posterior inferior parietal lobule, and left parahippocampal gyrus were less activated in individuals with PTSD. Although findings from their meta-analysis largely support the triple network model, the authors note that there were inconsistent findings with respect to the directionality of activation due to selection of comparison group, as well as reliable clusters of neural activation which were found outside of the anatomical demarcation of the central executive, salience, and default mode networks.^12^ Another compelling neurobiological model of PTSD is that of the dissociative subtype, first put forth by Bremner et al.^13^ In their neurobiological review of this model, Lanius et al.^14^ found that individuals with PTSD with chronic dissociation—as characterized by states of detachment, depersonalization, derealization, and subjective distance from their emotional experiences—contain unique neurobiological features, which can be distinguished from the non-dissociative sub-type of PTSD. Specifically, the dissociative subtype of PTSD was found to have a unique neural manifestation, as indicated by the overmodulation of midline prefrontal, dACC, and limbic regions of the brain.^14^

The traditional FNM has served as a crucial first step in understanding the neurobiology sub-serving anomalous affective processing in PTSD. However, the results of recent symptom provocation studies are inconsistent, thus suggesting that FNM may underrepresent the neurobiological complexity of PTSD. For example, Sartory et al.^15^ conducted a meta-analysis of 19 symptom provocation studies in individuals with PTSD. Their results provided partial support for the traditional neurocircuitry model, such that when comparing trauma-related stimuli to the control condition (e.g., neutral script), individuals with PTSD exhibited significantly greater activation in the bilateral amygdala, mid-line pregenual and retrosplenial cortices, as well as the occipital and angular gyri. However, support for the FNM of PTSD was not found when Satory et al.^15^ compared patterns of activation to trauma-related stimuli between individuals with PTSD and a trauma exposed control group (TEC). Instead, this between-subject contrast revealed that individuals with PTSD demonstrated greater activation of the mid-line retrosplenial cortex and precuneus in response to trauma-related stimuli. In a similar meta-analysis of 12 symptom provocation studies, Hayes et al.^16^ found that when comparing trauma-related stimuli to a neutral condition, individuals with PTSD demonstrated greater mid and dorsal ACC activation relative to a mixed control group (i.e., combination of TEC and non-trauma exposed controls (NTC)), which is consistent with the FNM; however, they also found hypoactivation of the precuneus. Finally, Ramage et al.^17^ conducted a meta-analysis of eight symptom provocation paradigms and found that relative to a mixed control group, individuals with PTSD had greater activation of the mid and posterior cingulate, as well as the precuneus. Taken together, the results of these meta-analyses have led to the growing supposition that the FNM, as tested by symptom provocation paradigms, may not fully represent the complex neurobiology underlying disrupted affective processing in PTSD.

In an attempt to expand beyond the FNM, studies of affective processing in PTSD have now been tested with a range of fMRI paradigms. For example, a review of recent neuroimaging meta-analyses focusing on affective processing in adult individuals with PTSD^15,16,18^ indicated the use of a diverse set of experimental paradigms, such as affective priming, backward masking, and tasks that employ non-trauma-related emotion stimuli. To date, only one study has reviewed the effect of affective paradigm type on neural activity in individuals with PTSD, though the authors selectively focused on the neural signature of symptom provocation studies and combined all other affective tasks with cognitive paradigms.^16^ As such, the first aim of this selective review is to identify task-related fMRI paradigms commonly used in studies of affective processing in individuals with PTSD. Next, we will classify these paradigms into a useful heuristic based on psychological conceptual principles, similarity of stimuli used, and fMRI task methodology. We then briefly describe the unifying principles of each category within the classification system, the experimental aim of the paradigm category and common methodological structure of these paradigms. Using this framework as a guide, we summarize the common and distinct patterns of neural activity that emerged within each paradigm classification. Considering its importance and proliferation in the neurobiological literature, we then compare these findings to the traditional FNM.^10^ Finally, we speak to several factors that may contribute to the heterogeneity of findings within the affective processing PTSD neuroimaging literature. This review selectively focuses on neuroimaging studies of affective processing conducted in adult individuals with PTSD and does not include neuroimaging literature produced from cognitive tasks which employ affective stimuli (e.g., emotional Stroop). The reader is referred to excellent comprehensive systematic reviews that have been recently published,^9^ as well as meta-analyses focusing on cognitive processing of affective stimuli,^16^ studies of symptom provocation,^15,17^ the neural signature of traumatic event type,^18^ and the effect of traumatic brain injury (TBI) on emotional and cognitive processing.^19^

## Study Identification and Selection

Using keywords “PTSD,” “neuroimaging,” “fMRI,” “PET,” “SPECT,” “affect,” and “emotion,” a literature search was conducted in PubMed, PsychInfo, and Google Scholar for neuroimaging studies of affective processing in adults with PTSD between the months of November and January of 2017 in order to identify comprehensive meta-analyses of affective processing in individuals with PTSD as compared to a control group (i.e., trauma exposed or non-trauma exposed control participants). This search yielded six meta-analyses conducted between 2012 and January of 2017.^12,15–19^ Of these meta-analyses, one was excluded as it examined the neural circuity of PTSD with and without mild traumatic brain injury.^19^ The remaining meta-analyses yielded 99 individual neuroimaging studies, of which 26 were redundant and removed, leaving 73 studies to review based on the following features: imaging modality (i.e., fMRI, PET, SPECT); target samples (e.g., PTSD vs. PTSD w/ comorbid personality disorder); comparison control group (i.e., trauma exposed controls, non-trauma exposed controls); criterion A traumatic event type (e.g., combat, motor vehicle accident); paradigm description and category (e.g., emotional trauma-related scenes, Go-No-Go task; emotion, cognitive, respectively); whole brain versus region of interest (ROI) analysis; hemodynamic response function (i.e., gamma, finite impulse response); and multiple comparison correction (e.g., Bonferroni, False Discovery Rate). Twenty-eight individual studies were eligible for inclusion (please note that two additional studies were found through review of references of meta-analyses), whereas 47 were deemed inappropriate for the scope of this review for the following reasons: fMRI paradigms were designed to assess cognitive functioning (e.g., episodic memory functioning) using affective stimuli;^20,21^ the samples included in the study were not relevant to the current review, such as adolescents with PTSD^22^ or the dissociative PTSD subtype only;^23^ the study assessed processing of physical pain as affective stimuli;^24^ the fMRI analyses were strictly correlational in nature;^25^ or the analyses were related to functional connectivity and not general linear models of task-related data.^23^ It should be noted that individual studies without standard comparison control groups (i.e., TEC, NT) were included; however, this was notated in results tables (i.e., those studies were not displayed with up or down arrows present). Finally, one article which met our criteria was included, as per the apropos suggestion of a reviewer,^26^ rendering a total of 29 studies included in the current review.

## “Symptom Provocation” Paradigms

These paradigms are referred to as symptom provocation, as they were originally theorized to be key in producing re-experiencing symptoms in individuals with PTSD.^8^ Within this classification, two distinct types of stimuli are routinely used: bespoke scripts of the traumatic event^7,23,25,27–39^ and trauma-related images, words, sounds, and smells.^26,40–43^

Script-driven imagery paradigms are the most commonly used fMRI paradigms in the neurobiological study of affective processing in PTSD^7,23,25,27–39^ (see Table 1). These techniques were first introduced into the PTSD literature by Pitman et al.^8^ The authors argued that standard combat-related stimuli (i.e., identical combat-related stimuli presented to all subjects) did not have the full capacity to reproduce uniquely stressful elements of an individual’s traumatic experience. The structure of script-driven imagery paradigms is largely standardized with respect to paradigm structure. Generally speaking, participants with PTSD are asked to describe a traumatic experience in as much sensory detail as possible. These descriptions are then condensed into a 30 to 40 s personalized script written in the second person, present tense. Typically, at least three script conditions are utilized in these paradigms: (1) a negative, traumatic experience; (2) a neutral non-traumatic every day experience; and (3) a baseline or recovery period. All scripts are audiotaped in a neutral voice and played back to each participant during a neuroimaging acquisition protocol. Procedurally, participants are typically instructed to listen carefully as their script is being read over the course of 30 s (i.e., Read Period). Next, they are then encouraged to recall as many sensory details that were associated with the traumatic event over the course of the next 30 s (i.e., Imagery Period). Next, participants enter a rest period for 120 s and instructed to “let go” of the traumatic memory (i.e., recovery period).

**Table 1.** Summary of whole brain and ROI-based symptom provocation studies in individuals with PTSD compared to NTC and TEC comparison groups.

Study	Method	PTSD (N)	NTC (N)	TEC (N)	Index trauma	Design	Control	Amy	Hipp	vm PFC	rACC	dACC	Insula
Whole brain analyses													
Bremner et al.^39^	PET	10	0	10	Combat	Event	Neutral			↓ R		↑ R	
Bremner et al.^27^	PET	10	0	12	SA	Block	Neutral		↓ R			↑ RL	↓ R
Britton et al.^29^	* PET	16	14	15	Combat	Block	Neutral	↓ L-N	↓ L-N		↓ R-NT		↓ RL-NT
Hopper et al.^24^	* fMRI	27	0	0	MVA + SA	Block	Neutral						
Lanius et al.^32^	fMRI	9	0	9	MVA + SA	Block	Implicit baseline			↓ RL	↓ RL		
Lanius et al.^31^	fMRI	7	0	10	MVA, PA, SA	Block	Implicit baseline			↓↑ R	↑ R		
Lanius et al.^34^	fMRI	10	0	10	MVA, PA, SA	Block	Implicit baseline			↓ L	↓ RL		
Lanius et al.^33^	* fMRI	11	0	13	MVA, SA	Block	Implicit baseline				↑ RL	↑ RL	↑ L
Lanius et al.^22^	* fMRI	21	0	10	MVA, PA, SA	Block	Implicit baseline			↑ L	↑ RL	↑ RL	↑ L
Rauch et al.^7^	PET	8	0	0	Combat, MVA, PA, SA	Block	Teeth clenched neutral	↑ R			↑ R		↑ R
Shin et al.^36^	PET	8	0	7	SA	Block	Teeth clenched neutral						
Shin et al.^37^	PET	8	0	8	SA	Block	Teeth clenched neutral			↓ R	↓ R	↓ R	
Shin et al.^38^	PET	17	0	19	Combat	Block	Neutral	↑ RL		↓ R	↓ L		
Vermetten et al.^25^	PET	8	0	8	Combat	Block	Neutral		↑ L		↑ L	↑ L	↑ RL
ROI analyses													
Lanius et al.^30^	* fMRI	26	0	16	MVA	Block	Implicit Baseline			↓ R	↓↑ RL	↑ RL	–
Liberzon et al.^40^	* PET	14	14	11	Combat	Block	White Noise	↑ L	–	↑ R			
Osuch et al.^35^	* PET	22	12	0	MVA	Block	Neutral	↓ R	↓ L	↓↑ L	–		↑ R
Pissiota et al.^41^	* PET	7	0	0	Combat, Torture	Block	Neutral	↑ R				↓ R-W	
Protopopescu et al.^42^	* fMRI	9	14	0	PA, SA	Block	Neutral	↑ L					

Note: W indicates activation was also found in whole brain analysis. Up-arrow indicates reported greater activation for PTSD group; down-arrow indicates reported deactivation for PTSD group. Patterns of activation are reported on statistically significant group-by-condition interactions. Cells without specified comparison groups indicate that the authors only reported within subject effects. Cells with – indicate a null effect, whereas empty cells mean that the effect was not tested. Statistical trend effects were not reported. MVA: motor vehicle accident; ND: natural disaster; PA: physical assault; SA: sexual assault; Amy: amygdala; Hipp: hippocampus; vmPFC: ventral medial prefrontal cortex; rACC: rostral anterior cingulate cortex; dACC: dorsal anterior cingulate cortex; L: left; R: right; N: non-trauma comparison group; T: trauma exposed comparison groups; NT: effect was found for both comparison groups; fMRI: functional magnetic resonance imaging; PET: positron emission tomography; ROI: region of interest; TEC: trauma exposed control group; NTC: non-trauma exposed control; PTSD: posttraumatic stress disorder.

* Authors reported conducting multiple comparisons correction.

Relative to script-driven imagery tasks, paradigms which employ trauma-related stimuli are the next most commonly utilized experimental approach to studying affective processing in individuals with PTSD^40–43^ (see Table 1). Similar to script-driven imagery approaches, these paradigms are designed to induce a particular PTSD symptom (e.g., re-experiencing) or emotion (e.g., anxiety). Trauma-related images that are germane to the traumatic event incurred by participants (e.g., images of collapsed coal mines presented to individuals whom had suffered a coal mining catastrophe), trauma-related sounds (e.g., machine gun firing, helicopters flying, explosions), or generic combat-related images (e.g., a man in fatigues holding a rifle) are the most commonly used type of stimuli within this category. Typically, these paradigms include a minimum of two conditions (i.e., trauma-related and neutral); however, some studies use a third condition of “rest” or baseline period. Procedurally, these paradigms are not standardized with respect to the presentation order or timing of the stimuli (i.e., block vs. event-related fMRI design), nor the use of a common control condition (e.g., neutral vs. a baseline rest period). Given the previous research conducted on symptom provocation studies,^15^ we would expect heterogeneous findings within this paradigm classification due to several methodological variables (e.g., comparison control group, statistical contrast map examined). However, recent research has suggested that individuals with PTSD may exhibit an over activated mid-line ACC when compared to control participants while processing trauma-related stimuli.^15^

**Figure 1.**
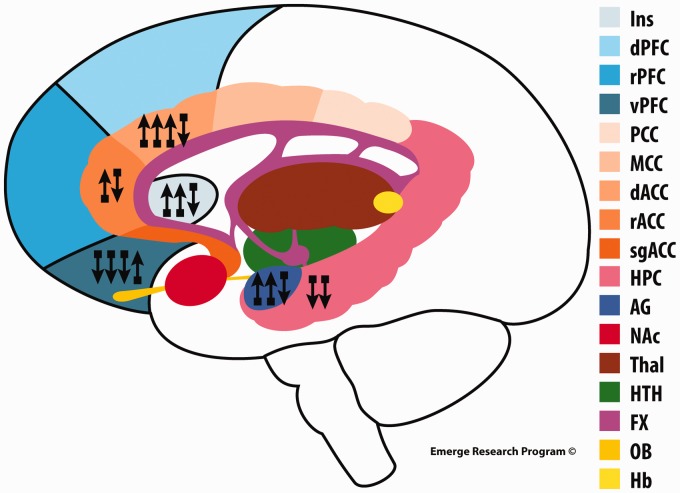
Patterns of hyper and hypoactivation in individuals with PTSD compared to TEC and NTC across studies using symptom provocation stimuli. Patterns of hyperactivation are denoted by up arrows, whereas hypoactivation are illustrated by down arrows. These patterns of activation are overlaid upon a right sagittal view of the brain, with colored areas representing brain regions commonly recruited during the neurobiological study of PTSD. Ins: Insula; dPFC: dorsol prefrontal cortex; rPFC: rostral prefrontal cortex; vPFC: ventral prefrontal cortex; PCC: posterior cingulate cortex; MCC: mid-line cingulate; dACC: dorsal anterior cingulate cortex; rACC: rostral anterior cingulate cortex; sgACC: subgenual anterior cingulate cortex; HPC: hippocampus; AG: amygdala; NAc: nucleus accumbens; Thal: thalamus; HTH: hypothalamus; FX: fornix; OB: olfactory bulb; HB: habenula.

Results from fMRI experiments using symptom provocation paradigms have been inconsistent and often contradictory (see Table 1 and Figure 1). With respect to amygdala activation, some studies reported amygdala activation in individuals with PTSD relative to no control group,^7,41,42^ whereas others reported hyperactivation of the amygdala relative to NTC group^42^ and hypoactivation relative to a mixed control group.^30,36^ Several studies under this classification demonstrated vmPFC hypoactivation in individuals with PTSD relative to a mixed control group,^31–33,35,36^ whereas some studies demonstrated the inverse relationship.^32,36^ Similar patterns were found for the rostral ACC, with studies demonstrating a trend towards hypoactivation relative to comparison groups.^30,31,33,35,38^ Relative to other regions implicated in the FNM of PTSD, there was a paucity of reported hippocampal findings under this paradigm classification,^30,36,40^ which is interesting given how these paradigms putatively rely on episodic memory.^8^

## Conscious Trauma-Unrelated Stimuli Paradigms

This classification of paradigms is referred to as conscious trauma-unrelated, as they employ stimuli taken from standardized stimulus sets and are not necessarily related to the traumatic event and are processed consciously.^44–48^ These paradigms are employed in order to explore patterns of neural activation related to specific emotion categories (e.g., fear, neutral), stimuli domain (e.g. faces, natural scenes), or a combination of the two (e.g., fearful faces, neutral objects). The most commonly utilized trauma-unrelated emotion stimuli are emotion faces, such as Ekman faces,^49^ NimStim standardized facial expressions,^50^ and images from the International Affective Picture System.^51^ Typically, these images are presented using a block design, in a pseudorandomized order of aversive, neutral, and baseline conditions, as the participant passively views the images,^44,47,48^ although a pseudo-randomized event-related designs have also been used.^45,46^ However, it should be noted that compared to other categories within the framework provided, the overall methodological structure of these paradigms is quite diverse with respect to several factors, such as stimulus selection and presentation length, control condition, as well as paradigm instruction. Results from previous literature employing such paradigms suggest that we would find a heterogenous pattern of neural activation within this class of studies; however, a recent review of the PFC role in emotion processing of such studies would suggest that the vmPFC would be hypoactivated in individuals with PTSD as compared to control participants.^52^

**Figure 2.**
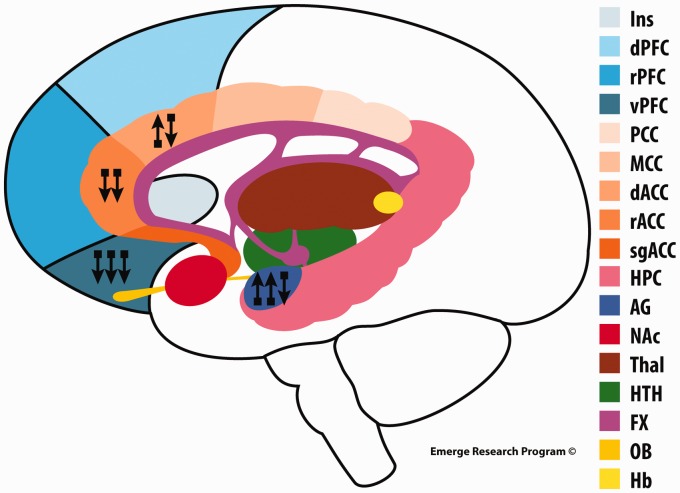
Patterns of hyper and hypoactivation in individuals with PTSD compared to TEC and NTC across studies using conscious trauma unrelated stimuli. Patterns of hyperactivation are denoted by up arrows, whereas hypoactivation are illustrated by down arrows. These patterns of activation are overlaid upon a right sagittal view of the brain, with colored areas representing brain regions commonly recruited during the neurobiological study of PTSD. Ins: Insula; dPFC: dorsol prefrontal cortex; rPFC: rostral prefrontal cortex; vPFC: ventral prefrontal cortex; PCC: posterior cingulate cortex; MCC: mid-line cingulate; dACC: dorsal anterior cingulate cortex; rACC: rostral anterior cingulate cortex; sgACC: subgenual anterior cingulate cortex; HPC: hippocampus; AG: amygdala; NAc: nucleus accumbens; Thal: thalamus; HTH: hypothalamus; FX: fornix; OB: olfactory bulb; HB: habenula.

Overall, these paradigms revealed a pattern of vmPFC deactivation in individuals with PTSD relative to TEC^47^ and NTC participants^46,48^ (see Table 2 and Figure 2). There was some evidence to suggest deactivation of the rostral ACC relative to TEC^47^ and NTC^48^ groups. Additionally, these paradigms demonstrated a pattern of amygdala hyperactivation relative to both TEC^47^ and NTC^48^ groups, as well as the deactivation of this region to both control groups.^46^ Finally, there was a dearth of significant findings in other regions of the traditional FNM, such as the hippocampus and dorsal ACC.

**Table 2.** Summary of whole brain and ROI-based conscious trauma-unrelated emotion studies in individuals with PTSD compared to NTC and TEC comparison groups.

Study	Method	PTSD (N)	NTC (N)	TEC (N)	Index Trauma	Study Design	Control	Amy	Hippo	vmPFC	rACC	dACC	Insula
Whole brain analyses													
Jatzko et al.^43^	* fMRI	8	8	0	Air show crash	Block	–						
New et al.^44^	* fMRI	14	14	14	SA	Event	Maintain					↓ RL-N	
ROI													
Phan et al.^45^	* PET	16	15	15	Combat	Event	Non-aversive	↓ L-NT		↓ L-N	–		–
Shin et al.^46^	fMRI	13	0	13	Combat, MVA	Block	Happy	↑ R		↓ L-W	↓ L-W	↑ L-W	
Williams et al.^47^	* fMRI	13	13	0	MVA, PA	Block	Neutral	↑ L		↓ RL	↓ RL		

Note: W indicates activation was also found in whole brain analysis. Up-arrow indicates reported greater activation for PTSD group; down-arrow indicates reported deactivation for PTSD group. Patterns of activation are reported on statistically significant group-by-condition interactions. Cells without specified comparison groups indicate that the authors only reported within subject effects. Cells with – indicate a null effect, whereas empty cells mean that the effect was not tested. Statistical trend effects were not reported. MVA: motor vehicle accident; ND: natural disaster; PA: physical assault; SA: sexual assault; Amy: amygdala; Hippo: hippocampus; vmPFC: ventral medial prefrontal cortex; rACC: rostral anterior cingulate cortex; dACC: dorsal anterior cingulate cortex; L: left; R: right; N: non-trauma comparison group; T: trauma exposed comparison groups; NT: effect was found for both comparison groups; fMRI: functional magnetic resonance imaging; PET: positron emission tomography; ROI: region of interest.

* Authors reported conducting multiple comparisons correction; TEC: trauma exposed control group; NTC: non-trauma exposed control; PTSD: posttraumatic stress disorder.

## Unconscious Trauma-Related and Unrelated Paradigms

Unconscious trauma-related and unrelated presentation paradigms present affectively laden stimuli outside of the participant’s conscious awareness; stimuli may be related to the traumatic event or not germane. Such paradigms are typically employed as a means of assessing the automaticity and unbiased neural response to trauma-related and neutral stimuli. Additionally, such paradigms reduce the contribution of cognitive process, as well as strategic responding.^53^ As such, these paradigms are thought to tap into more basic affective behavioral^54,55^ and neural responses.^56–58^ A review of the affective processing fMRI literature suggests that unconscious presentation paradigms are typically employed in two manners: affective priming^59,60^ and backward masking.^61–63^

Affective priming tasks investigate whether the unconscious evaluation of a primary stimulus (i.e., the prime) affects the conscious processing of a subsequent stimulus (i.e., the target).^64^ Commonly within fMRI PTSD research, these tasks present a prime stimulus that is affective in nature (e.g., sad faces, trauma-related stimuli) at a subliminal level (e.g., .15 s), followed by the rating of a neutral target stimulus (e.g., Chinese ideographs) presented at a supraliminal level (e.g., 1.85 s). Backwards masking paradigms test the phenomenon whereby the visibility of a target or primary stimulus is influenced by the presentation of a secondary stimulus.^65,66^ Within the affective processing PTSD literature, backward masking tasks typically present an affective visual stimulus of interest (e.g., trauma-related stimuli) briefly (e.g., 16.7 ms), which is followed within milliseconds by another visual stimulus (e.g., neutral scrambled), which effectively “masks” the effect of seeing the primary stimulus. The general methodological structure of unconscious presentation paradigms is quite homogenous and typically only varies with respect to the selection of stimulus used for the primary or secondary target. Neuroimaging research conducted in unconscious processing of affective stimuli would suggest that the amygdala would be consistently overrecruited in individuals with PTSD relative to control participants under this paradigm classification.^56,67^

**Figure 3.**
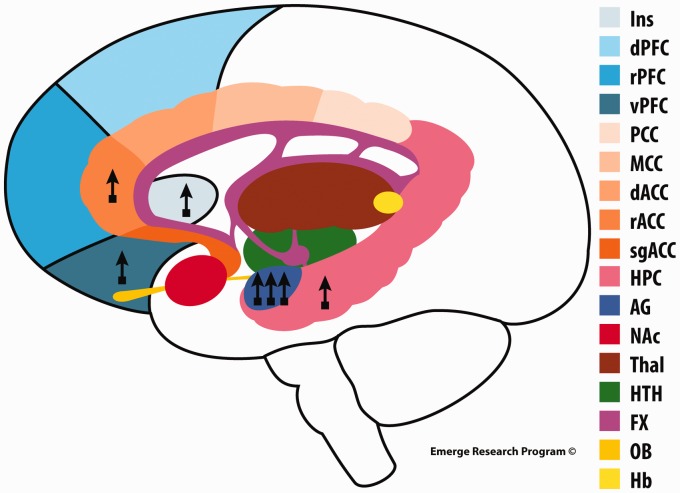
Patterns of hyper and hypoactivation in individuals with PTSD compared to TEC and NTC across studies using unconscious trauma related and unrelated stimuli. Patterns of hyperactivation are denoted by up arrows, whereas hypoactivation are illustrated by down arrows. These patterns of activation are overlaid upon a right sagittal view of the brain, with colored areas representing brain regions commonly recruited during the neurobiological study of PTSD. Ins: Insula; dPFC: dorsol prefrontal cortex; rPFC: rostral prefrontal cortex; vPFC: ventral prefrontal cortex; PCC: posterior cingulate cortex; MCC: mid-line cingulate; dACC: dorsal anterior cingulate cortex; rACC: rostral anterior cingulate cortex; sgACC: subgenual anterior cingulate cortex; HPC: hippocampus; AG: amygdala; NAc: nucleus accumbens; Thal: thalamus; HTH: hypothalamus; FX: fornix; OB: olfactory bulb; HB: habenula.

Several studies have sought to elucidate the neural mechanisms of PTSD via unconscious presentation paradigms^59–63,68^ (see Table 3 and Figure 3). Taken together, these paradigms routinely demonstrated that when unconsciously processing affective relative to neutral information, individuals with PTSD activated the amygdala to a greater extent when compared to trauma-exposed control participants,^60,63,68^ as well as to non-trauma exposed control participants.^59^ Indeed, only one unconscious presentation paradigm did not report amygdala hyperactivity in individuals with PTSD.^61^ Paradigms within this classification, however, did not consistently demonstrate hyper or hypoactivation of additional brain regions within the traditional neurocircuitry model of PTSD. For example, only one study demonstrated hyperactivity in the PTSD group of the left hippocampus^61^ relative to non-trauma exposed control participants.

**Table 3.** Summary of whole brain and ROI-based unconscious trauma-related and unrelated studies in individuals with PTSD compared to NTC and TEC comparison groups.

Study	Method	PTSD (N)	NTC (N)	TEC (N)	Index trauma	Study design	Control	Amy	Hippo	vmPFC	rACC	dACC		Insula
Whole brain analyses														
Mazza et al.^59^	* fMRI	10	10	0	–	Block	Fixation	↑ L						↑ R
Sakamoto et al.^60^	fMRI	16	0	16	PA, SA, Fire	Block			↑ L				↓	
ROI														
Bryant et al.^58^	* fMRI	15	0	15	MVA, PA	Block	Neutral	↑ RL		↑ RL	↑ RL			
Hendler et al.^67^	fMRI	10	11	0	Combat	Block	Scrambled	↑ R	–					
Rauch et al.^62^	* fMRI	8	8	0	Combat	Block	Happy	↑ R		–	–			

Note: W indicates activation was also found in whole brain analysis. Up-arrow indicates reported greater activation for PTSD group; Down-arrow indicates reported deactivation for PTSD group. Patterns of activation are reported on statistically significant group-by-condition interactions. Cells without specified comparison groups indicate that the authors only reported within subject effects. Cells with – indicate a null effect, whereas empty cells mean that the effect was not tested. Statistical trend effects were not reported. MVA: motor vehicle accident; ND: natural disaster; PA: physical assault; SA: sexual assault; Amy: amygdala; Hippo: hippocampus; vmPFC: ventral medial prefrontal cortex; rACC: rostral anterior cingulate cortex; dACC: dorsal anterior cingulate cortex; L: left; R: right; N; non-trauma comparison group; T: trauma exposed comparison groups; NT: effect was found for both comparison groups; fMRI: functional magnetic resonance imaging; PET: positron emission tomography; ROI: region of interest.

* Authors reported conducting multiple comparisons correction; TEC: trauma exposed control group; PTSD: posttraumatic stress disorder; NTC: non-trauma exposed control.

## Summary

Overall, we found that task-based fMRI paradigms largely fell into three coherent categories: symptom provocation, trauma-unrelated emotion, and unconscious presentation. In general, patterns of activation within each paradigm class revealed mixed results that cannot be solely attributed to task type. One exception to this general observation is the results of the unconscious presentation paradigms (Table 3 and Figure 3), which among all three paradigm classifications demonstrated the greatest reproducibility with respect to amygdala hyperactivation in individuals with PTSD, when compared to a mixed control group and control condition. This result was also underscored in a recent review focusing on the role of the amygdala in processing emotive and cognitive stimuli.^69^ Indeed, a number of neuroimaging studies have established the amygdala’s role in the automatic processing of affective stimuli.^56,58,63,70,71^ Importantly, unconscious presentation paradigms are often passive processing tasks, which have been shown to be a significant predictor of amygdala activation, as compared to tasks with explicit instruction^72^ (e.g., label the emotion of the stimuli). Furthermore, the amygdala is believed to be a key member of the salience network, an intrinsic connectivity network responsible for the detection and orientation to both internal, as well as external stimuli.^11^ As such, unconscious presentation paradigms may be particularly effective at eliciting differences in salience detection among individuals with PTSD, as compared to control participants. Paradigms which fell within the symptom provocation category exhibited a pattern of hyperactivation in the dACC and hypoactivation in the vmPFC in individuals with PTSD (Figure 1). Hyperactivation and hypoactivation were reported in other brain regions (Figure 1), though the directionality of results were not consistent across studies. Similarly, trauma-unrelated emotion paradigms revealed a pattern of vmPFC and rACC deactivation among individuals with PTSD, though results were not consistent across studies. Finally, as shown in Tables 1 to 3, we did not observe different patterns of findings from studies that included participants with different trauma types (e.g., combat vs. civilian trauma). Taken together, these results provide limited support for the FNM of PTSD within and across paradigm classification of affective processing studies.

The heterogeneity of results found in the aforementioned literature may be compounded by several biological and analytic factors. One such biological factor is related to the level of threat intensity associated with the traumatic event. Threat intensity refers to the propensity of a traumatic occurrence to lead to a lasting stress response.^73^ The threat intensity of the traumatic event is assessed across several domains, including severity, frequency, unpredictability, uncontrollability, and the inescapable nature of the traumatic event.^73–75^ Recent research has suggested that levels of threat intensity (e.g., mass shooting vs. 12-month active combat tour of duty) may be correlated with differential chronic stress pathology (CSP) burden, subsequently leading to varied patterns of biological abnormalities.^73–75^ Despite this and often in order to meet sample size and power requirements, neurobiological studies of PTSD tend to aggregate individuals into “mixed samples” and average CSP across different index traumas. Thus, larger study samples of individuals with PTSD may contain subsamples with differential threat intensities, CSP, and thus concomitant biological alterations. For example, recent research has suggested that CSP may be associated with functional and structural changes due to differential synaptic connectivity patterns.^75^ A dual pathology model was proposed, which highlights the possibility that trauma and stress may be associated with two distinct pathophysiological processes, that is, aminoacid-based pathology (ABP) versus monoamine-based pathology (MBP^76^). ABP is associated with treatment resistance to monoaminergic antidepressants, deficit in prefrontal and hippocampal gray matter, and dysregulation in glutamate and gamma-aminobutyric acid neurotransmission. In contrast, MBP is associated with enhanced response to monoaminergic antidepressants, gain in nucleus accumbens and basolateral amygdala gray matter, and dysregulation in serotonin and catecholamines.^75^ ABP is consistent with glutamate dysregulation and excitotoxicity, precipitating reduction in brain-derived neurotrophic factor (BDNF) and synaptic loss in the PFC and hippocampus. Specifically, ABP is hypothesized to disrupt glucocorticoid signaling, leading to increased neuronal inflammation, and impoverished astrocytic uptake of glutamate within the synapsis, resulting in extracellular glutamate and excitotoxicity. Conversely, MBP is thought to be related to disruption in norepinephrine and dopamine signaling leading to localized increase in BDNF and synaptic gain in the nucleus accumbens and basolateral amygdala.^75^ As such, this model suggests that individuals with ABP-based pathology may have differential patterns of neuronal firing, as compared to individuals with a predominate MBP-based pathology, thus potentially contributing to the heterogeneity found in the PTSD neuroimaging literature.

With respect to analytic factors, a whole brain versus ROI approach may significantly contribute to the heterogeneity of findings within this literature. For example, approximately half of the studies included in this selective review used amygdala ROI analyses, of which 83% reported activation. Conversely, only three of the remaining studies which did not employ a ROI analysis reported amygdala activation. Although we contend that ROI analyses are useful for restricting analyses to specific brain regions and controlling for Type I error by limiting the number of statistical tests, this analytic approach is not without limitation.^77^ For example, Sprooten et al. conducted a meta-analysis on the results of studies which employed task-fMRI data in a range of psychiatric disorders (i.e., schizophrenia, major depressive disorder, bipolar disorder, anxiety disorders, and obsessive-compulsive disorder) and found that on a region-by-region basis, ROI studies accounted for the over-representation of the amygdala and caudate nucleus activation, which was not supported when whole-brain studies were considered. These findings suggested that the a priori selection of a ROI, in addition to the neuroimaging fields’ resistance against publishing negative results, may lead to the over-simplification and over-localization of psychiatric neurobiological models. Future studies exploring the neurobiological underpinnings of PTSD symptomology may choose to move beyond an ROI approach and use alternative statistical methodologies (e.g., multi-voxel pattern analysis) to explore large-scale distributed sub-cortical to cortical networks.^78^ For example, multi-voxel pattern analysis techniques allow for neuroimaging data to be reduced to highly reproducible special patterns of activity through a supervised classification classifier.^79^

The choice of comparison group and contrast condition may be additional analytic factors contributing to contradictory patterns of neural activity within this literature. The selection of a comparison group is often contingent upon paradigm design. For example, researchers using a script-driven symptom provocation design often employ a TEC group, as the nature of the experimental design requires that the control group have exposure to a traumatic event. Whereas other design types, such as unconscious presentation paradigms, often use comparison groups that are naïve to trauma (i.e., NTC). To address the relevance of comparison group, Patel et al.^12^ conducted two separate meta-analyses in individuals with PTSD relative to TEC and NTC groups. The results of their study revealed that those with PTSD exhibited amygdala hyperactivation relative to NTC and not TEC groups, thus indicating that this pattern of activation may be, at least partially, a neural marker of trauma exposure and not pathophysiology of PTSD per se.^12^ Relatedly, there is variability with respect to selection of a consistent control condition. For example, script-driven symptom provocation studies demonstrated the use of a generic neutral and “teeth clenching” neutral^37,38^ conditions, as well as the use of an implicit baseline control condition (i.e., fixation condition, wherein the participant is staring at a fixation) when performing statistical contrasts.^31–35^

Finally, variable statistical thresholding approaches to data analysis may be yet another analytic factor contributing to divergent neuroimaging results of affective processing in PTSD. We concede that many of the studies included in this review were conducted in the nascent stages of the neuroimaging field and before specific guidelines of statistical reporting were specified.^80–82^ That being said, we observed a variety of liberal statistical thresholds when addressing multiple comparisons corrections. For example, a number of the studies reviewed here did not report a correction for multiple comparisons (see Tables 1, 2, and 3), used a fixed effects statistical model, liberal cluster-based inference correction (e.g., *p* < 0.05; 3 voxel cluster extent), or employed an arbitrary voxel-based inference correction (e.g., *p* < 0.001) when reporting their findings. Although we acknowledge that several of these studies are exploratory in nature and therefore amenable to liberal thresholding, we recommend that future confirmatory studies adhere to current minimal statistical standards.^80–83^

## Limitations

A chief limitation of the current review is the availability of studies which met our criteria for inclusion and our reliance on studies which were included in recent meta-analyses. Although our original search criteria identified five meta-analyses which met criteria, resulting in 99 eligible studies, further inspection deemed nearly half of these ineligible, largely due to the utilization of a cognitive paradigm structure. As such, we acknowledge that the scope of this review narrowly focuses on affective processing paradigms conducted in individuals with PTSD. Relatedly, another limitation of the current review is in our utilization of the FNM as a framework for assessing results of the aforementioned affective studies, as the FNM was formulated upon the results of a multitude of studies which used diverse stimuli and a variety of paradigm structures (e.g., conditioning and extinction, episodic memory, inhibition, passive viewing, affective processing).

## Conclusions and Future Directions

The neural systems subserving affective processing in individuals with PTSD have been examined using several experimental designs over the last two decades. A selective review of this literature found that affective tasks coalesce into a useful heuristic with three categories: symptom provocation, trauma-unrelated emotion, and unconscious presentation. Although neural patterns of activation remain heterogeneous, we did observe that unconscious presentation paradigms were superior in eliciting amygdala hyperactivation in individuals with PTSD relative to a comparison control group. This finding offers partial support for the FNM of PTSD, though we contend that this model may be lacking in its ability to account for the neurobiological complexity of the disorder. Although several biological and analytic factors can account for a portion of heterogeneity, future research remains warranted to advance our understanding of the neurobiological mechanisms governing affective processing in PTSD. The results of our qualitative review, in addition to the findings from quantitative meta-analyses containing affective processing studies, suggest the need for a better understanding of trauma-related, as well as analytic variables involved in interpreting emerging patterns of neural signal. Further investigations are warranted for analyses which take these factors into greater consideration with respect to their role in neural alterations associated with PTSD.
